# Synthesis, Photophysical Characterization and Evaluation of Biological Properties of C7, a Novel Symmetric *Tetra*-Imidazolium-*Bis*-Heterocycle

**DOI:** 10.3390/microorganisms11020495

**Published:** 2023-02-16

**Authors:** Hannah Kunstek, Melaine Wang, Hiba Hussein, Ines Dhouib, Bassem Khemakhem, Arnaud Risler, Stephanie Philippot, Celine Frochot, Philippe Arnoux, Bertrand Fournier, Mihayl Varbanov, Florence Dumarçay-Charbonnier

**Affiliations:** 1 Université de Lorraine, CNRS, L2CM, F-54000 Nancy, France; 2 Graz University of Technology, 8010 Graz, Austria; 3 Sorbonne Université, 75005 Paris, France; 4 Laboratory of Plant Biotechnology, Sfax Faculty of Sciences, BP 1171, University of Sfax, Sfax 3038, Tunisia; 5 LRGP UMR7274 CNRS-UL, 54000 Nancy, France; 6 Institut Galien Paris-Saclay, CNRS UMR 8612, Université Paris-Saclay, 91400 Orsay, France; 7 Université Paris-Saclay, CentraleSupélec, CNRS, Laboratoire SPMS, 91190 Gif-sur-Yvette, France; 8 Laboratoire de Virologie, Centres Hospitaliers Régionaux Universitaires (CHRU) de Nancy Brabois, 54500 Vandœuvre-lès-Nancy, France

**Keywords:** *bis*-imidazolium-pyridine, photophysical characteristics, biological properties, antibacterial, antiviral

## Abstract

A novel symmetric *tetra*-imidazolium-*bis*-heterocycle, called C7, was designed and synthesized in a quick two-step pathway, with the objective to synthesize biologically active supramolecular assembly. The synthesized compound was then analyzed for its photophysical properties, for a potential application in theragnostic (fluorescence) or phototherapy (photodynamic therapy, with the production of reactive oxygen species, such as singlet oxygen ^1^O_2_). C7 was thus screened for its biological activity, in particular against important human pathogens of viral origin (respiratory viruses such as adenovirus type 2 and human coronavirus 229E) and of fungal and bacterial origin. The compound showed limited antiviral activity, combined with very good antiproliferative activity against breast cancer, and head and neck squamous cell carcinoma models. Interestingly, the selected compound showed excellent antibacterial activity against a large array of Gram-positive and Gram-negative clinically isolated pathogenic bacteria, with a possible inhibitory mechanism on the bacterial cell wall synthesis studied with electron microscopy and molecular docking tools. Collectively, the newly synthesized compound C7 could be considered as a potential lead for the development of new antibacterial treatment, endowed with basic photophysical properties, opening the door towards the future development of phototherapy approaches.

## 1. Introduction

N-heterocyclic carbenes (NHCs) have been gaining research attention over years [1], as they tend to have great photosensitizing properties [2,3], due to their structural features [4,5]. The NHCs can form stable complexes and are therefore used in the development of photoactive molecular systems, which can then be applied in phototherapy (PT) [6,7,8,9]. PT has been shown to be quite effective and is commonly utilized in treating multiple bacterial and viral pathologies [10,11,12,13]. Therefore, our research focuses on the synthesis and structural modulation of NHC complexes and supramolecules that would target pathogens responsible for causing human diseases, with a radiation-enhanced efficacity. The objective of our recent project was to synthesize biologically active supramolecular assemblies with macro-N-*bis*-heterocyclic *bis*-imidazolium structures [14,15], which could be used as photosensitizers in PT. As the synthesis of NHC tends to be challenging, we decided to test each complex at the time, and modify it according to the obtained data. In this paper, we present the biological characterization of the compound C7—more specifically, the evaluation of the anti-infectious properties of the molecules in absence of activating irradiation. Qualification and metabolic tests were conducted with the goal of revealing the mode of action of synthesized molecules. Additionally, photophysical, physicochemical and structure-activity properties were assessed. The molecular structure described here (C7), bearing two *bis*-imidazolium-pyridine units linked by two 7-methylene chains, has been shown by Gaussian calculations to have a favorable octahedral geometry for preparing transition metal complexes. Calculations on transition metal complexes based on ligands with shorter methylene chains exhibit strong distortion of their *bis*-imidazolium-*bis*-pyridine units. Indeed, many Fe(II) complexes have been described in the literature with similar but non-cyclic structures [2]. These compounds have good photophysical and luminescence properties, but these complexes do not have a long enough lifetime, especially for applications in phototherapy [3]. It is therefore necessary to rigidify their structure in order to increase their stability and thus their lifetime. However, only a few studies have focused on the preparation of structures combining both a macrocycle and an imidazolium, and on their metal coordination complexes. Among them, macrocyclic *tetra*-N-heterocyclic carbenes (NHC) ligands have been reported with structures similar to porphyrins, showing better electronic transfers to the transition metal due to the presence of the NHC units [16,17]. We therefore chose to prepare macrocyclic structures bearing two symmetrical chains of seven carbons with the aim of preparing octahedral transition metal complexes with UV-visible absorption lengths in the near-infra-red spectrum and a longer lifetime. Such properties are of major importance in the design of new photosensitizers. It is important to note that the C7 macrocycle is completely soluble in aqueous medium which allowed us to study the biological properties of the free molecule.

## 2. Materials and Methods

### 2.1. Products

Synthesized macrocyclic carbene ligand (Table 1) C7 was dissolved in distilled water to obtain the required final concentration.

### 2.2. Bacteria and Culturing Conditions

Microorganisms (Table 2) were cultured overnight at 35 °C in Mueller Hinton Broth, Cation-Adjusted (BD, BD 212322, New York, NY, USA) and adjusted to 0.5 McFarland (standard providing an optical density comparable to the density of a bacterial suspension with a 1.5 × 10^8^ colony forming units (CFU/mL)), according to standardized guidelines NF EN ISO 20776-1 [18]. 

### 2.3. Cell Lines and Culture Conditions 

Cells (Table 3) were cultured according to The Global Bioresource Center, ATCC recommendations [19]. All the cells were incubated at 37 °C with 5% CO_2_. 

### 2.4. Preparation of Cells for SEM

Bacteria of the exponential growth phase in Luria–Bertani Broth (LB) medium were treated accordingly with sub-minimal inhibitory concentration, or sub-MICs, of C7 (2 µg/mL) for 8 h at 35 °C. Untreated controls were prepared in standard LB medium. The bacteria were then fixed with 30% ethanol, and the samples were dehydrated with graded ethanol series. All reagents used were electron microscopy (EM) grade and purchased from Delta Microscopies (France). Microscopy was performed with a Hitachi S-4800 microscope. Secondary electron images were taken at low electron energies between 1 keV and 2.5 keV.

### 2.5. Antibacterial Properties Assays

The minimal inhibitory concentration (MIC) test was performed according to the standardized guidelines NF EN ISO 20776-1 [18]. Once MIC was obtained, a bactericidal test was performed. Briefly, 10 µL from wells at 1X, 2X, 4X and 8X MIC were spotted for counting and incubated 18 h +/− 2 h at 35 °C on Mueller Hinton Agar (BD, 225250, New York, NY, USA) in triplicat. No growth on spots at <=4X MIC shows that the product is bactericidal (>3log10 CFU reduction at <= 4X MIC compared to the initial concentration of 5.105 CFU/mL). Experiments were repeated three times.

### 2.6. Antifungal Properties Assay 

Antifungal properties were tested on *Candida glabrata,* cultured in liquid Sabouraud medium at 35 °C, according to the standardized protocol EUCAST E.DEF 7.3.2 [20]. Maximal concentration tested was 16 µg/mL. Itraconazole (ThermoFisher 452870050, ThermoFisher Scientific, Waltham, MA, USA), well-known as a broad-spectrum fungicide, was included in the assay as a control which was then compared with tested samples [21]. Once we obtained MIC, a fungicidal test was performed similarly as the bactericidal test on Sabouraud Agar, according to the EUCAST standardized protocol [20]. 

### 2.7. Protein Leakage Test

Molecules at chosen concentrations were incubated with *E. coli* (0.5 MIC = 4 µg/mL; MIC = 8 µg/mL, 2 MIC = 16 µg/mL) and *S. epidermidis* (0.5 MIC = 2 µg/mL; MIC = 4 µg/mL, 2 MIC = 8 µg/mL) at 0.5 McFarland, for 3 h at 35 °C. Upon incubation, the supernatant was collected and filtered (0.2 µm), and the Bradford assay was performed according to the manufacturer’s instructions (Bradford Dye Reagent #5000205). 

### 2.8. Cytotoxicity Assay

Cytotoxicity of products was tested on MRC-5, MCF-7, SCC-25, and Vero cells with a seeding concentration of 10,000–15,000 cells/well. Products were diluted with 2% FSC MEM media (SIGMA, M4655, Missouri, USA) to concentrations ranging from 1 to 256 µg/mL and incubated with 80% confluent cells for 24 h. Then, 72 h after product addition, the 3-[4,5-dimethylthiazol-2-yl]-2,5 diphenyl tetrazolium bromide (MTT) assay was performed for 2 h according to established laboratory protocol, as previously described [22], and optical density was measured at 540 nm. 

### 2.9. Cytopathogenicity Assay

MRC-5 and Vero cells were seeded at a concentration of 10,000 cells/well and 15,000 cells/well respectively and incubated overnight to reach 80% confluence. Products of interest were diluted in the 2% FCS MEM media at concentrations lower than IC80 (maximal 20% cell inhibition). Furthermore, human coronavirus (hCoV229E) and human adenovirus 2 (AdV2) were diluted 10-fold up to 10^7^ (1 to 10,000,000), (Table 4). Virus dilutions were made with prepared product–media dilutions. Cells were washed with PBS and 10% FCS MEM was replaced with virus–product–media after which cells were incubated for 72 h at 33 °C, in the case of coronavirus infection. On day 3 of incubation, crystal violet staining was performed according to the established laboratory protocol, previously described [23]. 

### 2.10. Hemolysis Assay

Hemolysis assay was performed according to the established protocol [22], where blood collected at CHU Nancy was washed three times with PBS, and 10^7^ cells/mL were incubated for 30 min at 37 °C with prepared products. Products were previously diluted in a range from 0.5 to 150 µg/mL with PBS. As a positive control, cells were resuspended with 0.01% polysorbate 20 (Tween 20). Upon incubation, samples were centrifuged for 5 min at 800× *g*, and the supernatant was collected for optical density reading at 540 nm. The blank was deducted, and the obtained values were normalized to a positive control (0.01% Tween). 

### 2.11. Antiproliferation Assay

Antiproliferation properties of ligand were tested on MCF-7 and SCC-25 cells (10,000 cells/well) in a range from 1 to 256 µg/mL for 72 h. Upon incubation, MTT assay was performed.

### 2.12. Synthesis

Synthesis of the precursor 1: 2,6-di(1H-imidazol-1-yl) pyridine: the compound has been described in the literature [24]. A large Schlenk tube fitted with a stirring rod is charged with one equivalent of 2,6-dibromopyridine (2.0 g, 0.0085 mol, 2.0 eq.), imidazole (3.47 g, 0.051 mol, 6.0 eq.) and potassium carbonate (4.65 g, 0.037 mol, 4.0 eq.) without solvent. The reaction mixture was degassed and placed under argon atmosphere. Then the mixture was stirred at 190 °C for 18 h. After cooling to room temperature, the mixture was taken up in a minimum of water, extracted three times with CH_2_Cl_2_ (ca. 25 mL) and washed three times with saturated aqueous Na_2_CO_3_ solution. The organic phases are collected, dried over MgSO_4_ and filtered. The filtrate is evaporated under reduced pressure to give 1.30 g of a light beige powder (75%).

**NMR ^1^H**  (400 MHz, CDCl_3_): δ 8.39 (t, ^4^J = 1.62 Hz, 2H, H_2′_), 7.97 (t, ^3^J = 7.9 Hz, 1H, H_4_), 7.68 (t, 2H, ^3^J = 1.96 Hz, 2H, H_4′_), 7.30 (d, ^3^J = 7.9 Hz, 2H), 7.24 (t, ^3^J = 1.68 Hz, 2H, H_5′_)

**NMR ^13^C**  (100,6 MHz, CDCl_3_): δ 148.5, 142.2, 135.1, 131.3, 116.2, 109.7

Synthesis of compound C7: (12Z,32Z,112Z,132Z)-11H,31H,111H,131H-2,12(2,6)-dipyridina-1,3,13(1,3),11(3,1)-tetraimidazol-3-iumacycloicosaphane-13,33,113,133-tetraium bromide can be briefly described hereafter. In a Schlenk reactor conditioned under argon, 0.200 g (0.94 mmol; 1 eq.) of 2,6-bis(imidazolyl)pyridine and 6-7 mL of anhydrous acetonitrile were introduced. Later, 0.16 mL (0.94 mmol; 1 eq.) of 1,7-dibromoheptane was also added to the suspension, which was then heated to reflux for 7 days at 82 °C. The reaction progress was monitored by thin layer chromatography (TLC) in a saturated acetone/H_2_O/KNO_3_ eluent (10/3/1). The obtained precipitate was filtered, washed with acetonitrile, recovered with a few milliliters of water, and freeze-dried to give 0.355 g of a white lyophilized powder of the C7 macrocycle in a 40% yield.

**NMR ^1^H (400 MHz, D_2_O):**  δ 9,85 (s, 4H, H_2′_); 8.36 (t, ^3^*J* = 8.1 Hz, 4H, H_3_); 8.30 (s, 4H, H_5′_); 7.94 (d, ^3^*J* = 8,1 Hz, 2H, H_4_); 7.75 (s, 4H, H_4′_); 4.35 (t, ^3^*J* = 7.2 Hz, 8H, H_α_); 1.97 (p, ^3^*J* = 7.0 Hz, 8H, H_β_); 1.39 (bs, 12H, H_γ,_ H _δ_).

**NMR ^13^C (100.6 MHz, D_2_O):**  δ 145.62 (C_2_); 144.77 (C_2′_); 123.63 (C_4_); 123.58 (C_4′_); 119.63 (C_5′_); 114.77 (C_3_); 50.50 (C_γ_); 27.57 (C_δ_); 25.27 (C_β_); 25.15 (C_α_).

**SM (MALDI)**  : m/z = 615.467 [M-4Br-3H]^+^

**Figure 1:**
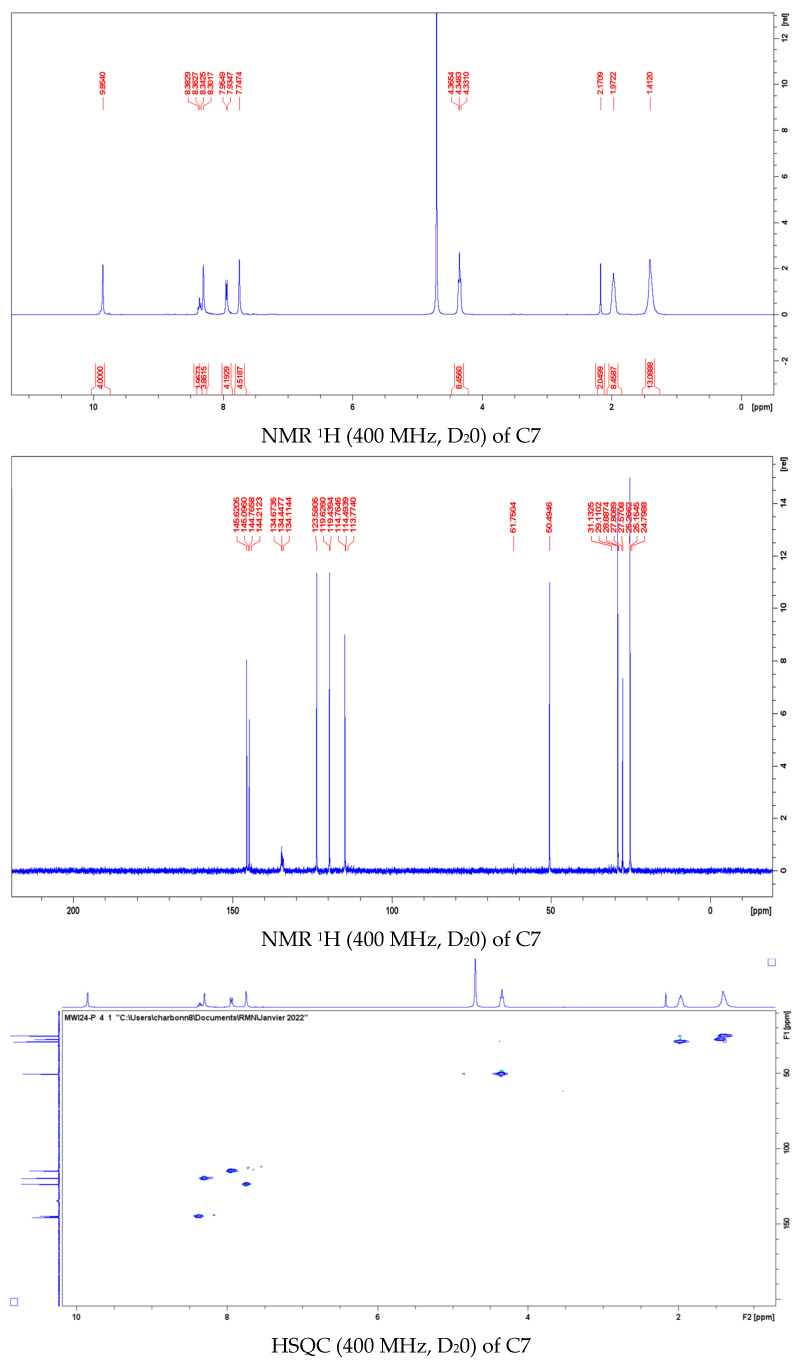

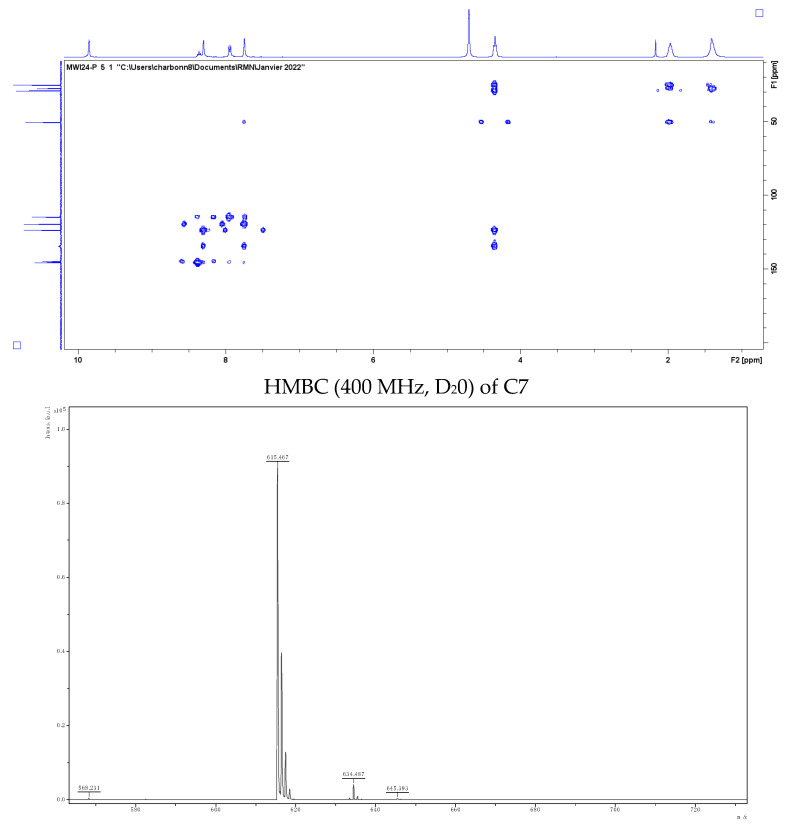
Mass spectrum of C7 MALDI.

MS analysis performed in positive mode on a MALDITOF ultraflex III (bruker) using a HCCA matrix (α-Cyano-4-hydroxycinnamic acid) (Figure 1).

**Figure 2:**
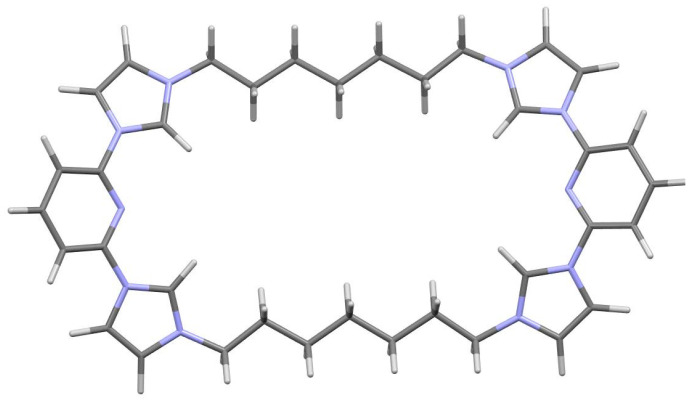
Structure optimization of C7 by DFT/B3LYP/6-31G*.

The ligand structure has been optimized in a vacuum using DFT/B3LYP/6-31G* method (Figure 2).

### 2.13. Photophysical Analysis

UV-Visible-Fluorescence Spectrophotometry-Singlet Oxygen Production: absorption spectra were recorded on a UV-3600 UV-visible double beam spectrophotometer (SHIMADZU, Marne La Vallée, France). Fluorescence spectra were recorded on a Fluorolog FL3-222 spectrofluorimeter (HORIBA Jobin Yvon, Longjumeau, France) equipped with a 450 W Xenon lamp, a thermostatically controlled cell compartment (25 °C), an R928 UV-visible photomultiplier (HAMAMATSU Japan) and an InGaAs infrared detector (DSS-16A020L Electro-Optical System Inc, Phoenixville, PA, USA). 

The excitation beam is diffracted by a SPEX double-lined grating monochromator (1200 grooves/mm blazing at 330 nm), and the emission one by a SPEX double grating monochromator (1200 grooves/mm blazing at 500 nm). Singlet oxygen emission was detected by a SPEX double grating monochromator (600 grooves/mm blazing at 1 µm) and a long wave pass (780 nm). All spectra were measured in 4-sided quartz cuvettes. All emission spectra (singlet oxygen fluorescence and luminescence) were displayed with the same absorbance, with lamp and photomultiplier correction. 

The fluorescence quantum yield is defined as the ratio of the number of photons emitted over the entire fluorescence spectrum divided by the number of photons absorbed. It is of course independent of whether the fluorescence spectrum is represented in the wavelength scale or in the wavenumber scale. 

The fluorescence quantum yield was calculated from Equation (1):

(1) $$\phi_{f}=\phi_{f_{0}}.\frac{I_{f}}{I_{f_{0}}}.\frac{DO_{0}}{DO}.{\left(\frac{n}{n_{0}}\right)}^{2}$$

where $\phi_{f}$ and $\phi_{f_{0}}$, $I_{f}$ and $I_{f_{0}}$, *DO* and *DO*_0_, *n* and *n*_0_ are the quantum yield, fluorescence intensities, optical densities and refractive index of the sample and the standard respectively. 

The standard used is the quinine sulphate in 0.5 M H_2_SO_4_ with $\phi_{f_{0}}$ of 0.55139.

Fluorescence quantum yields are usually determined by integration of the fluorescence spectrum. Indeed, the fluorescence intensities $I_{f}$ and $I_{f_{0}}$ correspond to the areas found of the fluorescence curves of the sample and the reference. The optical densities *DO* and *DO*_0_ correspond to the absorbance values obtained at a wavelength λ = 290 nm for the sample and the reference.

### 2.14. Molecular Docking

The three-dimensional crystal structure of Penicillin Binding Protein (PBP) from *Streptococcus pneumoniae* (pdb code: 1QME) was downloaded from Protein Data Bank in pdb format with resolution 2.4 Å [25]. It was then prepared with AutodockVina tools. All water, ligand molecules, and solvent molecules were removed, and hydrogen atoms were added before being saved in pdbqt format to be included as a target protein in the virtual screening. The binding pocket was identified with a co-crystallized ligand (cefuroxime). Then, the grid box center was adjusted X = 4182, Y = 36.022 and Z = 16.251 with dimensions for PBP, and its size was set to 66 × 42 × 44 Angstroms to cover the active site. Cefuroxime was redocked in the structure to validate the docking process. Molecular docking analysis (binding types, binding energies, inhibition activities, distances, and possible interactions) was performed by the AutodockVina program between a synthetic molecule and PBP [26]. Docking results were examined using Drug Discovery Studio and LigPlot (v.20.1.0.19295) and Viewer Lite.

## 3. Results and Discussion

### 3.1. Antibacterial and Antifungal Properties

C7 showed overall good antibacterial activity when tested on several clinically relevant bacterial isolates (Table 2)—mostly being bactericidal in the range of 4–16 µg/mL. The best activity was displayed for *S. epidermidis*. Additionally, C7 turned out to be fungistatic for *C. glabrata* with the activity displayed in the range of 1.28–5.12 µg/mL (Table 5). 

### 3.2. Protein Leakage 

**Figure 3:**
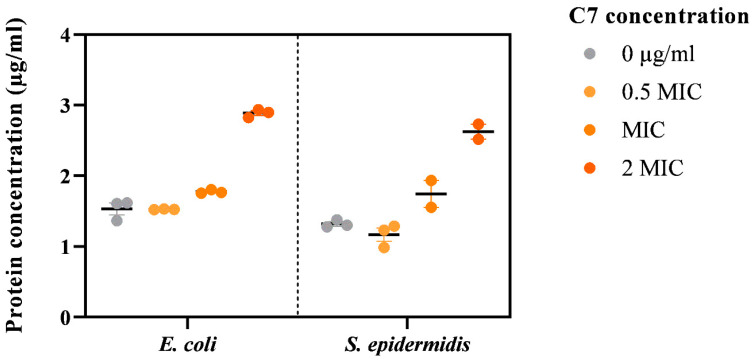
Effect of C7 on *E. coli* and *S. epidermidis* membrane (*n* = 3).

Generally, there were more proteins found in supernatants of *E. coli* samples in comparison to *S. epidermidis* samples. The increase in protein leakage was correlated with the increase in product concentration (Figure 3). Protein leakage could suggest the membrane impairment caused by the product upon incubation. However, further experiments are needed to investigate the exact mechanism of action of synthesized ligand. 

### 3.3. Molecular Docking Assay

Penicillin-binding proteins (PBP), periplasmic membrane-attached proteins, crucial for maintaining the bacterial cell wall [25] and one of the most well-known targets for antibacterial drugs, could also be affected by the compound C7. In previous studies, morphological changes in the surface of bacteria, such as the presence of altered or irregular shapes in bacteria treated with antibiotics, correlate with the performance of these antibiotics against penicillin-binding proteins (PBPs) [27,28]. According to these studies, penicillin-binding proteins (PBP) could be a target for our molecule C7. In order to predict the validities of this hypothesis, an in silico study was released. 

**Figure 4:**
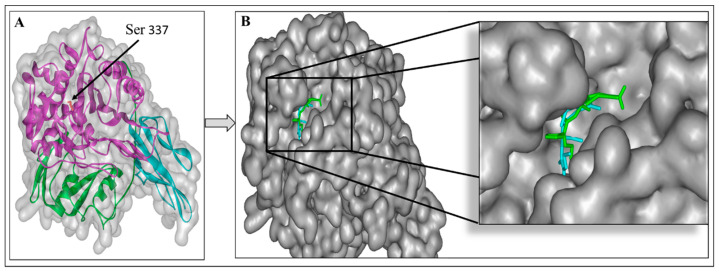
3D structure of Staphylococcus pneumoniae Penicillin-binding protein (PBP) (pdb code: 1QME); N-terminal (blue), C-terminal (green), and central domain (pink) and catalytic residues Ser 337 (red, stick) (**A**). The superimposed cefuroxime position in the active site of PBP of the solved 3D structure (blue) and re-docked structure (green) (**B**).

Structurally, the PBP has three domains—the N-terminal, C-terminal, and central domain. The central domain, also named the transpeptidase domain, contains the catalytic serine 337. The active site is located in a groove that passes through the transpeptidase domain [29] (Figure 4A).

To predict the inhibitory potency of the molecule C7 and standard compound cefuroxime with PBP as the target, a docking study was carried out. First, the native co-crystal inhibitor (cefuroxime) was re-docked in order to validate the entire docking process and ensure its reproducibility. The result revealed that this native ligand’s re-docking occupies the same active site of the PBP as the co-crystal ligand does in the original docking structure (PDB ID: 1QMF) (Figure 4B). The re-docked ligand’s binding energy at the active site of PBP is −8.0 Kcal/mol. The obtained value is in accordance with the literature, the binding energy of cefuroxime is −7.54 Kcal/mol as described by Sarojin et al., in 2014 [30]. 

**Figure 5:**
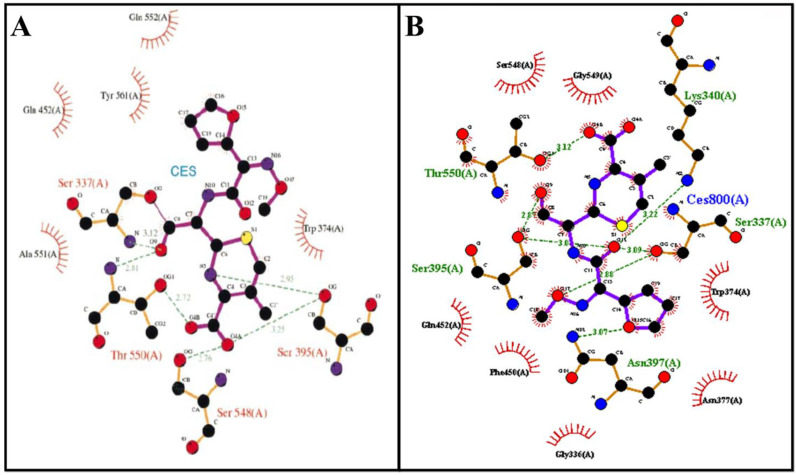
Binding mode of the cefuroxime (**A**) from literature and redocked (**B**). Adapted from Gordon, E et al., 2000 [25].

In addition, the re-docking showed that cefuroxime interacts with many similar residues to the literature, including Ser 584, Ser 337, Trp 374, Thr 550, and Gln 452 (Figure 5A,B).

**Figure 6:**
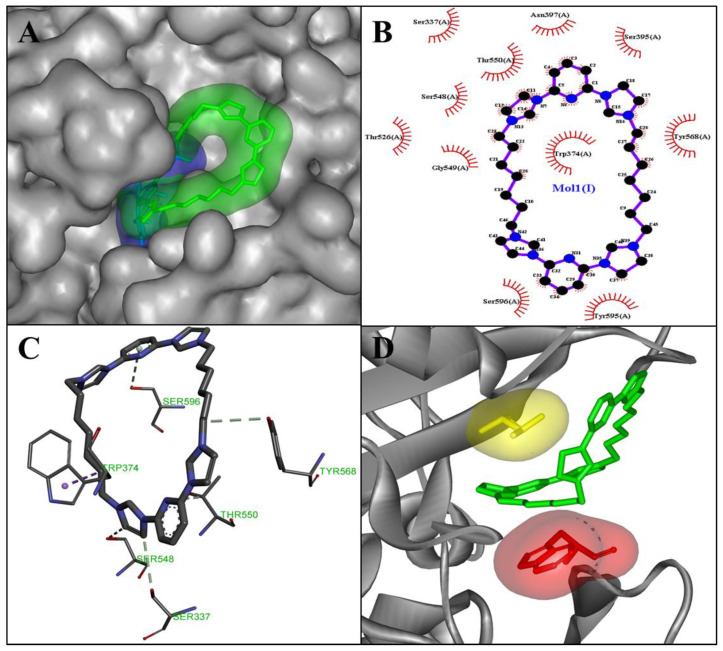
A close-up surface view of the superimposed cefuroxime (blue) and molecule C7 (green) (**A**); interaction result of docked C7: 2D view (**B**) and 3D view (**C**). Visualization of the hydrophobic stacking interactions made by C7 with Trp 374 (red) and Thr 550 (yellow) (**D**).

Once the docking was validated, the molecule C7 has been docked to PBP. According to the binding energy, molecule C7 exhibited a high binding affinity (−12.7 kcal/mol) compared to the control ligand (cefuroxime). The obtained value was higher than that measured by Mir et al. (2022). In fact, the highest binding energy to PBP was measured for elatine at −9.2 kcal/mol [31]. The binding energy is directly related to the stability of the complex formed between the ligand and the target protein [31]. To explain the high binding energy of C7, we analyzed the binding pose of C7 in PBP active site, and the interactions established between this ligand and the protein target at the active site. The overlay of the stander ligand (cefuroxime) crystal structure with the C7-PBP docking complex shows that C7 binds at and covers most of the active site of PBP (Figure 6A). The docking study revealed that C7 interacts with 10 different amino acids overall; Ser 337, Trp 374, Ser 395, Asn 397, Thr 526, Ser 548, Gly 549, Thr 550, Tyr 568, Tyr 595, and Ser 569. Four carbon–hydrogen bonds were established with Ser 337, Ser 548, Thr 550, and Tyr 568 amino acids. In addition, hydrophobic interaction, i.e., π-σ was created between the compound C7 and Trp 374 (Figure 6B,C). Furthermore, this compound makes hydrophobic stacking interactions with Trp 374 and Thr 550 which enhance the C7 binding to the active site of PBP (Figure 6D). Interestingly, these results showed that compound C7 interacts with significant amino acids Ser 337 (catalytic amino acid), Trp 374 and Tyr 568 as designated by Mir et al., (2022) [31]. According to the results found by Damasceno, J.P.L., in 2017, C7 also established interactions with key amino acids Ser 395, Ser 548, and Thr 550 [28]. Thus, this compound can be used further as an antibacterial drug by inhibiting cell wall synthesis.

### 3.4. Electron Microscopy Assay

**Figure 7:**
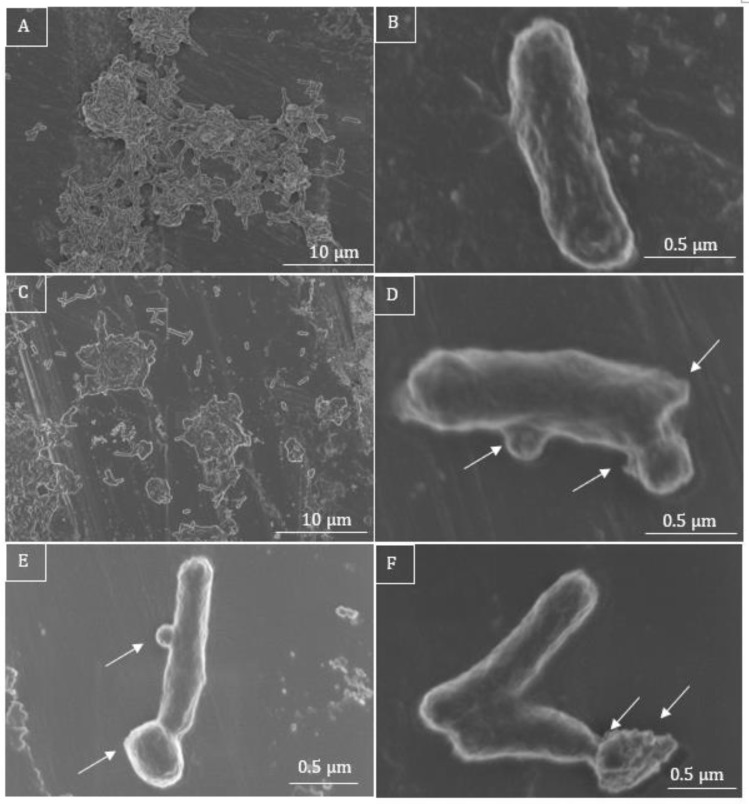
SEM micrographs of *E. coli*. Untreated bacteria are long, intact, and evenly shaped (**A**,**B**), whereas C7-treated bacteria are rather clustered (**C**) and present irregular shapes and surface corrugated (**D**–**F**). When incubated with a sub-MIC of 2 µg/mL (**D**–**F**), the cells show blisters on their surface, close to the polar and septal regions, as well as small protruding bubbles on their surface (**D**–**F**, white arrows).

The untreated *E. coli* cells displayed a smooth and intact surface (Figure 7A,B). After incubation with sub-MICs (2 µg/mL) of C7, however, the bacteria significantly increased their compactness, and showed a tendency to form clusters (Figure 7B). Multiple blisters of different shapes on the bacterial surface were observed after incubation with the sub-MIC of C7. The latter concentration induced the protrusion of numerous small bubbles of a few nanometers in size, which could be seen only in regions not covered with platinum (Figure 7D–F).

The surface of the bacteria in the medium with C7 looked corrupted, but their average length seemed to remain unchanged (Figure 7B,D–F).

**Figure 8:**
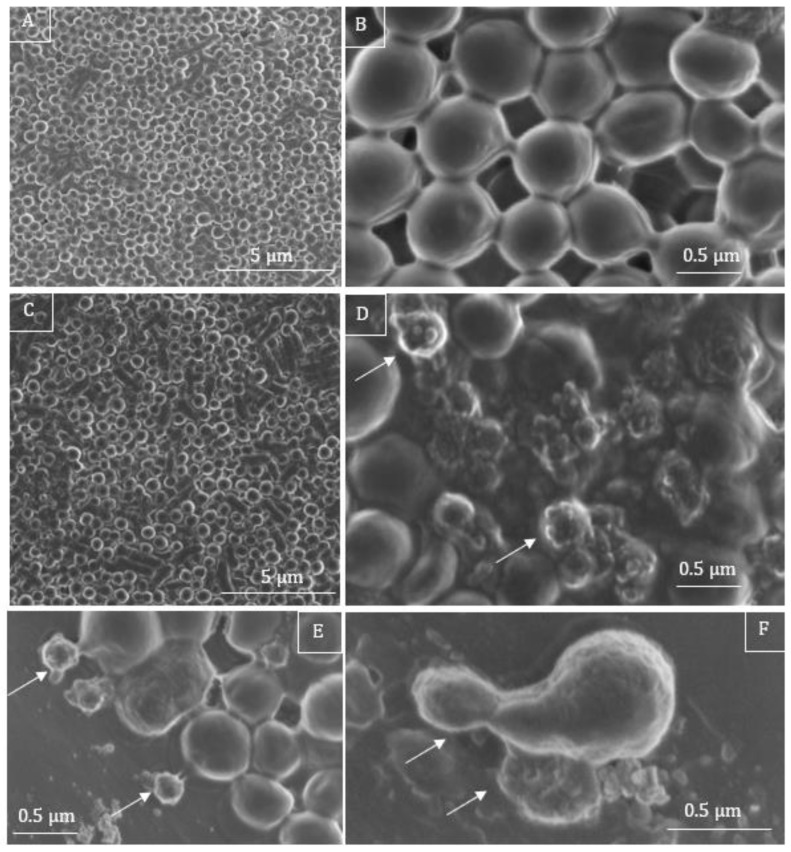
SEM micrographs of *S. aureus*. Untreated bacteria are round and intact (**A**,**B**). After treatment with C7 at a sub-MIC of 2 µg/mL bacteria present irregular shapes (**C**,**F**), partially irregular cellular surface (**D**–**F**), accompanied by burst cells and completely lysed cells are found, with damaged cell wall and caused the non-uniform distribution of cytoplasmic materials (**D**–**F**), white arrows.

In the control samples of *S. aureus* in LB medium without treatment, the cocci looked round and undamaged (Figure 8A,B). The incubation with a sub-MIC of C7 (2 µg/mL), led to the lysis of bacteria (Figure 8D–F), where some bacteria had burst with numerous lysed cells in clusters. Irregularly shaped bacteria were also observed (Figure 6F), as well as shrunken cells, and cells with damaged membrane, in the close presence of cell debris were also noted (Figure 8D–F, white arrows).

### 3.5. Cytotoxicity Assay

Inhibitory concentration (IC50) of C7 was calculated at 13.7 µg/mL for MRC-5 cells and at 6.9 µg/mL for Vero cells (Table 6). C7 seemed to be more cytotoxic for Vero cells than the MRC-5 cells. 

### 3.6. Antiproliferation Assay

**Figure 9:**
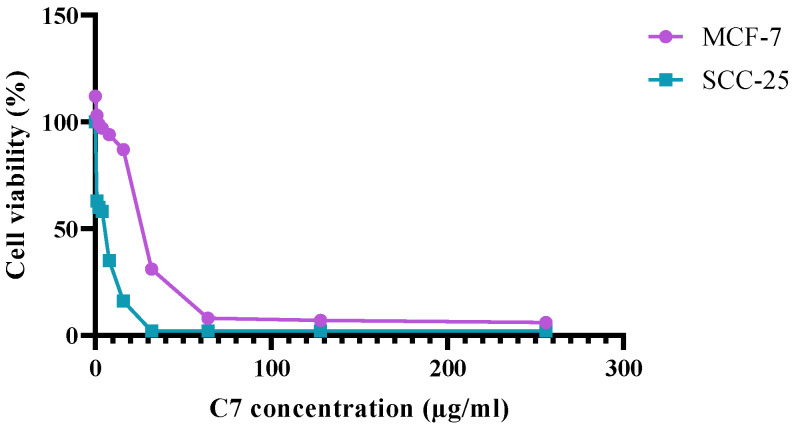
Effect of C7 on human breast cancer (MCF-7) and human skin cancer (SCC-25) cells.

Data suggests that C7 can drastically reduce both MCF-7 and SCC-25 cancer cells viability, with 50% of inhibition at 39.9 µg/mL for MCF-7 and 4 µg/mL for SCC-25 cells (Figure 9). 

### 3.7. Cytopathogenicity Assay 

**Figure 10:**
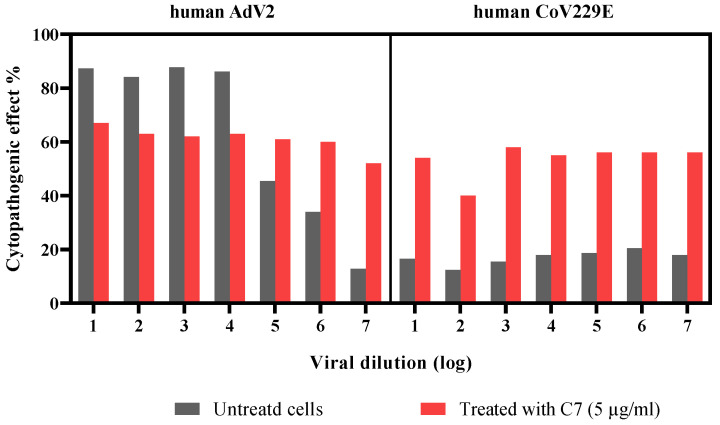
Product activity on human adenovirus-2 (hAdV2) and coronavirus 229E (hCoV229E) at different viral loads.

A high cytopathogenic effect was observed when Vero cells were infected by either hAdV2 or hCoV229E and treated with C7. The product concentration used for the assay (5 µg/mL) was too high, which explains the visible cytotoxic effect. Despite the high cytotoxic effect, there was no viability improvement noted at lower viral loads in comparison to untreated samples (Figure 10). 

### 3.8. Hemolysis 

**Figure 11:**
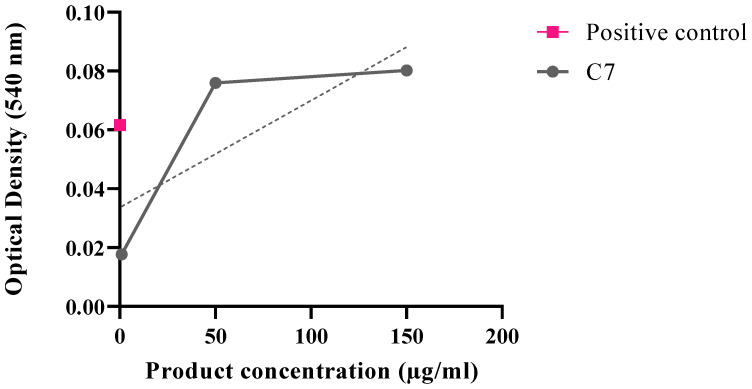
Optical density measured upon product incubation with human blood cells.

In comparison to a positive control (cells incubated with 0.01% Tween 20), C7 showed an increase in human red blood cells cytotoxicity, especially at higher product concentrations (Figure 11). Furthermore, there was an increasing hemolytic tendency noted. 

### 3.9. Synthesis

**Figure 12:**
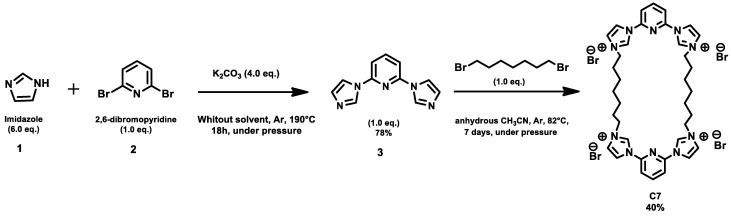
Chemical synthesis pathway of C7.

Compound C7 (Table 1) was synthesized in two steps (Figure 10). The first step is the di-halopyridine reaction which consists of an aromatic nucleophilic substitution at high temperature and without solvent to give intermediary product **3** in 78% yield [24]. This reaction is carried out with a large excess of imidazole (6.0 equivalents) and in the presence of potassium carbonate under argon at 190 °C for 18 h. The second step is a second order nucleophilic substitution reaction of 1.0 equivalent of the compound **3** with 1.0 equivalent of the dibromoheptane derivative in anhydrous acetonitrile, under argon and reflux for 7 days to give the pure product in 40% yield (Figure 12).

### 3.10. Photophysical Analysis

**Figure 13:**
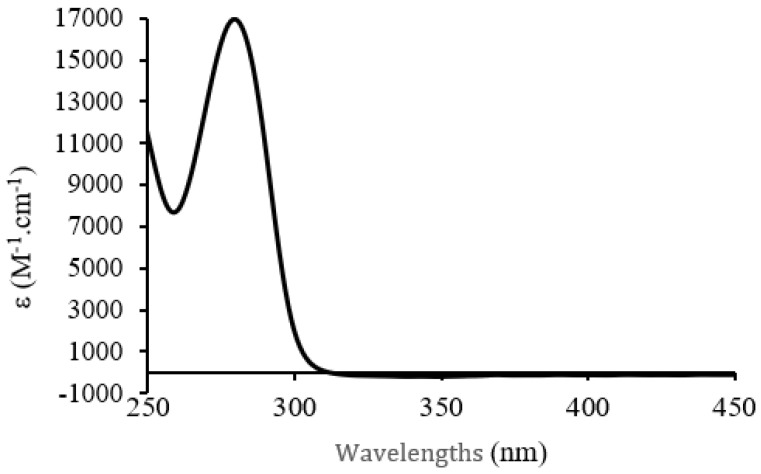
UV-visible absorption spectrum of C7 in D_2_O C = 1 × 10^−3^ mol·L^−1^.

The absorption spectrum in D_2_O shows an intense absorption maximum in the UV region at 290 nm, characteristic of the π-π* ligand-centered (LC) transition (Figure 13).

**Figure 14:**
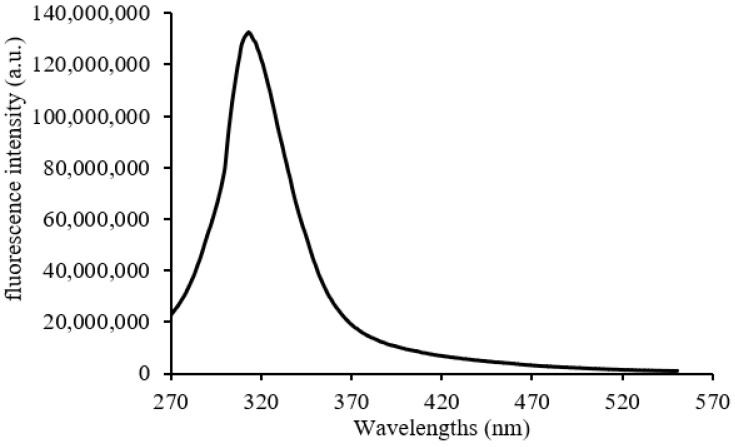
Luminescence emission spectra of C7 in D_2_O. C = 5 × 10^−5^ mol·L^−1^.

Concerning the luminescence spectra of C7 in D_2_O excitation at 290 nm causes luminescence emission of the molecule at 314 nm (Figure 14).

**Figure 15:**
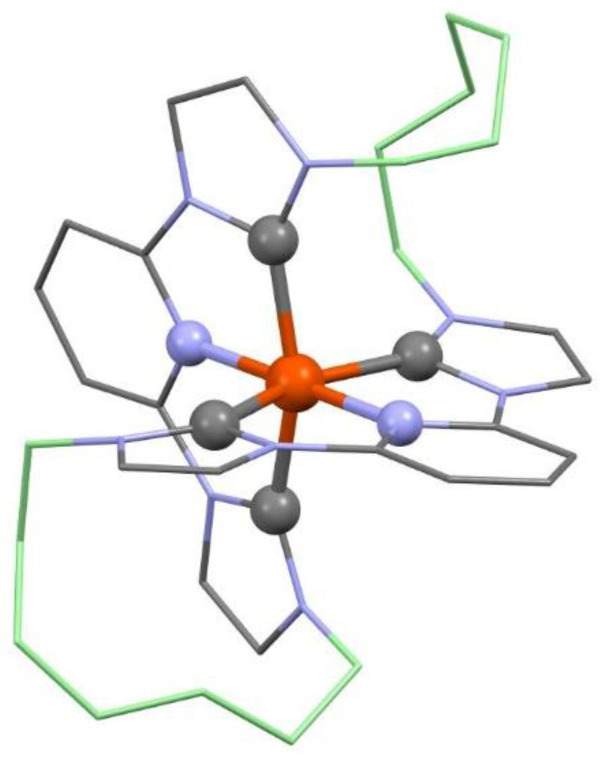
Structure optimization of C7-Fe(II) *in vacuum* by DFT/B3LYP/6-31G*. Hydrogen atoms are omitted and the two 7-methylene chains colored in light green for clarity.

The C7 compound unfortunately showed no singlet oxygen production and only 10% fluorescence quantum yield, which does not make it a good photosensitizer candidate. Our objective is to prepare new compounds with photophysical properties more suitable for phototherapy applications with respect to the photosensitive molecules already used and described in the literature. It is therefore necessary that the synthesized compound presents a light absorption in the near infrared without light degradation and leading to singlet oxygen production. Various transition metals can be considered to achieve this, particularly iron which is an abundant and inexpensive metal. The challenge then lies in stabilizing the C-metal bond and having a sufficiently long life. However, Gaussian calculation predicts a favorable octahedral coordination site for a potential C7-Fe complex, which justifies future endeavors for the complexation of the C7 ligand with Fe or other metals (Figure 15).

## 4. Conclusions

In this paper we describe the original synthesis and the photophysical characteristics (NMR, MALDI-TOFF, UV-visible, fluorescence) of a novel water-soluble symmetric *tetra*-imidazolium-*bis*-heterocycle C7 with good yield for putative application in anti-infectious phototherapy (aiPT). The compound has shown interesting antiproliferative activity against cancer cell line models such as MCF-7, in a three-dimensional model for the study of human breast cancer, and SCC-25, used as an established model for head and neck squamous cell carcinoma. The intrinsic cytotoxicity of C7 against cancer cells was also observed against normal human fibroblasts and red blood cells, indicating that the right balance between anticancer and spearing healthy cells and an appropriate targeting for specific administration to the cancer cells is yet to be found. The antiviral activity tested with respiratory non-enveloped human adenovirus type 2 and enveloped human coronavirus 229E, was limited. Nevertheless, C7 showed promising antifungal and antibacterial activity, against a large array of clinical bacterial isolates, including Gram-negative and Gram-positive pathogens, being bactericidal at concentrations as low as 5 µg/mL. The exploration of the putative mechanism of action suggests that C7 damages the membrane of the bacteria, with a possible impact on the synthesis of the cell wall, in the case of Gram-positive bacteria, leading to protein leakage, loss of cell integrity, and finally to cell lysis. The limited photoactivity of the compound, with the absence of ^1^O_2_ and the low fluorescence quantum yield, discard the possibility of its direct use in phototherapy approaches, such as photodynamic therapy, suggesting that further modifications in its structure may be needed, possibly by the complementation with metal ions (Fe, Cu, Au, etc.).

## Figures and Tables

**Table 1:** Synthesized heterocyclic carbene ligand structure, absorbance data.

Structure	Product Name	Chemical Formula	Product Specifications
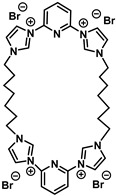	C7	C_36_H_46_Br_4_N_10_	Absorbance: 290 nm Emission: 320 nm

**Table 2:** Origin and Gram-staining information of experimental microorganisms.

Genus	Species	Origin	Year	Gram-Staining	Abbreviation
*Acinetobacter*	*calcoaceticus*	Clinical isolate, CHU Nancy	2022	negative	Ac
*Escherichia*	*coli*	ATCC 25922	2021	negative	Ec
*Enterococcus*	*faecalis*	Clinical isolate, CHU Nancy	2021	positive	Ef
*Klebsiella*	*pneumoniae*	Clinical isolate, CHU Nancy	2021	negative	Kp
*Pseudomonas*	*aeruginosa*	ATCC 27853,	2021	negative	Ps
*Staphylococcus*	*aureus*	ATCC 29213,	2020/21	positive	Sa
*Staphylococcus*	*epidermidis*	ATCC 14990,	2020	positive	Se
*Candida*	*glabrata*	Clinical isolate, CHU Nancy	2021/22	-	Cg

**Table 3:** Origin information of experimental cells.

Cell Line		Origin
MRC-5	Lung fibroblasts	ATCC CCL-171
MCF-7	Breast cancer cell line	ATCC HTB-22
Vero	Epithelial kidney cells	ATCC CCL-81
*SCC-25*	Squamous cell carcinoma	ATCC CRL-1628

**Table 4:** Origin information of experimental viruses.

Virus	Abbreviation	Origin
Human coronavirus 229E	hCoV229E	ATCC VR-740
Adenovirus 2	AdV2	ATCC VR-846

**Table 5:** Summary of antibacterial and antifungal properties of C7 (*n* = 2, with 8 replicates per condition, in accordance with the standardized protocols).

Microorganism	C7 MIC	Antimicrobial Effect
*Acinetobacter calcoaceticus*	4–16	bactericidal
*Escherichia coli*	4–16	bactericidal
*Enterococcus faecalis*	4–16	bactericidal
*Klebsiella pneumoniae*	4–16	bactericidal
*Staphylococcus aureus*	4–16	bactericidal
*Staphylococcus epidermidis*	2–8	bactericidal
*Candida glabrata*	1.28–5.12	fungistatic

**Table 6:** Inhibitory concentration of C7 for MRC-5 and Vero cells (*n* = 2).

	MRC-5	Vero
**IC50**	13.7 µg/mL	6.9 µg/mL

## Data Availability

Not applicable.
